# Identifying novel biomarkers of the pediatric influenza infection by weighted co-expression network analysis

**DOI:** 10.1186/s12985-019-1231-8

**Published:** 2019-10-29

**Authors:** Mohadeseh Zarei Ghobadi, Sayed-Hamidreza Mozhgani, Mahdieh Farzanehpour, Farida Behzadian

**Affiliations:** 1 Department of Virology, School of Public Health Tehran University of Medical Sciences, Tehran, Iran; 2 Institute of Biochemistry and Biophysics, University of Tehran, Tehran, Iran; 3 Department of Microbiology, School of Medicine, Alborz University of Medical Sciences, Karaj, Iran; 4 Non-communicable Diseases Research Center, Alborz University of Medical Sciences, Karaj, Iran; 5 Department of Bioscience and Biotechnology, Malek Ashtar University of Technology, Tehran, Iran

**Keywords:** Influenza, Co-expression network, Biomarker, Pathogenesis

## Abstract

**Background:**

Despite the high yearly prevalence of Influenza, the pathogenesis mechanism and involved genes have not been fully known. Finding the patterns and mapping the complex interactions between different genes help us to find the possible biomarkers and treatment targets.

**Methods:**

Herein, weighted gene co-expression network analysis (WGCNA) was employed to construct a co-expression network among genes identified by microarray analysis of the pediatric influenza-infected samples.

**Results:**

Three of the 38 modules were found as the most related modules to influenza infection. At a functional level, we found that the genes in these modules regulate the immune responses, protein targeting, and defense to virus. Moreover, the analysis of differentially expressed genes disclosed 719 DEGs between the normal and infected subjects. The comprehensive investigation of genes in the module involved in immune system and viral defense (yellow module) revealed that *SP110*, *HERC5*, *SAMD9L*, *RTP4*, *C19orf66*, *HELZ2*, *EPSTI1*, and *PHF11* which were also identified as DEGs (except *C19orf66*) have the potential to be as the biomarkers and also drug targeting for the treatment of pediatric influenza.

**Conclusions:**

The WGCN analysis revealed co-expressed genes which were involved in the innate immune system and defense to virus. The differentially expressed genes in the identified modules can be considered for designing drug targets. Moreover, modules can help to find pathogenesis routes in the future.

## Background

Influenza virus is one of the most incident infectious agent, which is mainly classified into three types of A, B, and C. The prevalence amount of type A is more than other influenza types in the world. Also, the burden of seasonal influenza virus caused the infection of 3–5 million cases with severe illness symptoms [1]. Influenza viruses affect the human life more than other respiratory illnesses. The pathogenesis of Influenza has not been yet well understood since it depends on the immune system and viral determinants. Moreover, the previous infection or vaccination causes the cellular immunity which affected the efficacy of infection with various seasonal, zoonotic, and pandemic influenza viruses [2]. Therefore, different hosts can have distinct effects on the incidence level of the disease.

Microarray is a high-throughput technique has the ability of simultaneous measuring of thousands of gene expressions and so generating tremendous data. In order to general and then detailed evaluation of the biological phenomena in each study, the special and sometimes complicated statistical analysis is required [3, 4].

Many genes are involved in the pathogenesis routes of influenza infection, which constitute the complicated networks. Among various genes, the change in expression levels of some genes regulate the expression of others, so they can put in a group called module. These modules have different biological responsibility. Also, each group may activate various pathways and control specific functions [5]. Finding the modules can help researchers to design the proper treatment route by targeting key proteins with specific functions. Moreover, finding novel biomarkers including important functions in a pathogenic-related disease is of importance for prognosis, risk assessment, and progression monitoring of disease [6, 7]. Biomarkers can be detected by calculation of differentially expressed genes between the normal and infected subjects, however, discovery the proteins that each biomarker is in connection with them, help to find the key pathways functioning in a disease.

Exploration of the involved genes which have close correlation patterns could be achieved by weighted gene co-expression network analysis (WGCNA) [8]. Through this algorithm, the highly co-expressed genes are placed in one module and different modules may contain key genes implicated in the pathogenesis process [9].

In this study, we aimed to find modules containing highly co-expressed genes which involve in the pediatric influenza pathogenesis. Also, the key genes with the prognosis potential were identified. Moreover, the genes in each module were analyzed using Gene Ontology (GO) function and pathway enrichment methods.

## Methods

### Gene expression data and preprocessing

The microarray gene expression dataset GSE29366 was downloaded from the NCBI Gene Expression Omnibus (GEO). It contains 12 healthy subjects and 19 Pediatric influenza patients which their whole blood samples were analyzed. All of the involved children in this study were in the age range from 1.5 months to 17 months. All individuals developed primary infection and showed signs of the disease. The Illumina HumanWG-6 v3.0 expression beadchip (GPL6884) was used to produce the sequencing data. The goodSamplesGenes function in Weighted Gene Co-expression Network Analysis (WGCNA) was used to filter the samples and genes with too many missing entries and zero-variance genes [10].

### Weighted gene co-expression network

The weighted gene co-expression network based on Pediatric influenza samples was constructed by R package “WGCNA” [11]. To this purpose, the adjacency matrix containing Pearson’s correlations between each gene pair was firstly generated with the optimized soft power and then was transformed into a Topological Overlap Matrix (TOM). The highly co-expressed genes were then grouped by hierarchical clustering. In the next step, the dynamic tree cut algorithm was utilized to cut clustering dendrogram branches and generation of the modules. The gene expression profiles of each module were summarized by the first principal component named as module eigengene (ME). Moreover, each module denotes as discrete colors. Finally, the similar modules (module eigengenes) were merged into a single module and was then used to further interpretation [12].

### Finding differentially expressed genes

To detect the differentially expressed genes (DEGs) among normal and infected subjects, GEO2R tool in the GEO database was employed. Benjamini-Hochberg FDR- adjusted *p*-values < 0.05 was selected as a criterion for finding DEGs.

### Finding candidate genes and their functional annotations

The top highly interconnected genes in each module were identified as hub genes. Afterward, they were submitted in the Search Tool for the Retrieval of Interacting Genes (STRING) database to build the protein-protein interaction network (PPIN) [13]. Then, the nodes with zero betweenness and degree one were set aside from the constituted network. The PPIN was visualized using Gephi 0.9.2 [14]. The gene ontology and pathway enrichment analysis were carried out in g:profiler website (https://biit.cs.ut.ee/gprofiler/) [15].

## Results

### Data preprocessing and sample selection

**Fig. 1:**
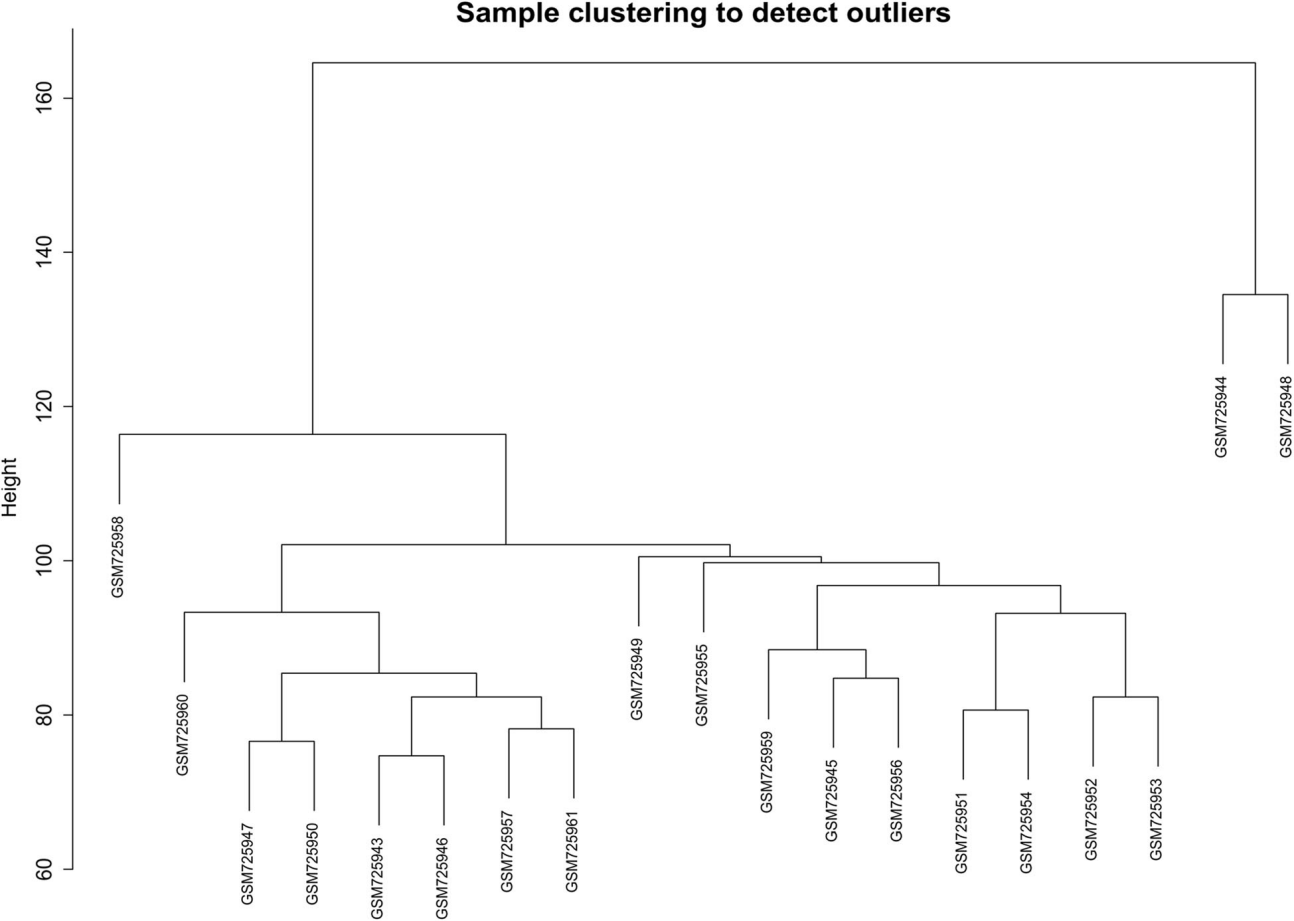
Hierarchical cluster tree of samples

Gene expression dataset GSE29366 was firstly quantile normalized and log-transformed. Then, the probe sets with unknown gene name were removed. The samples were evaluated in terms of missing entries and zero-variance genes. The number of 15,436 probes remained from 28,742 probes. As shown in the cluster tree (Fig. 1), two samples (GSM725944 and GSM725948) are clustered in different branches and excluded for further analysis.

### Weighted gene co-expression network construction and modules identification

**Fig. 2:**
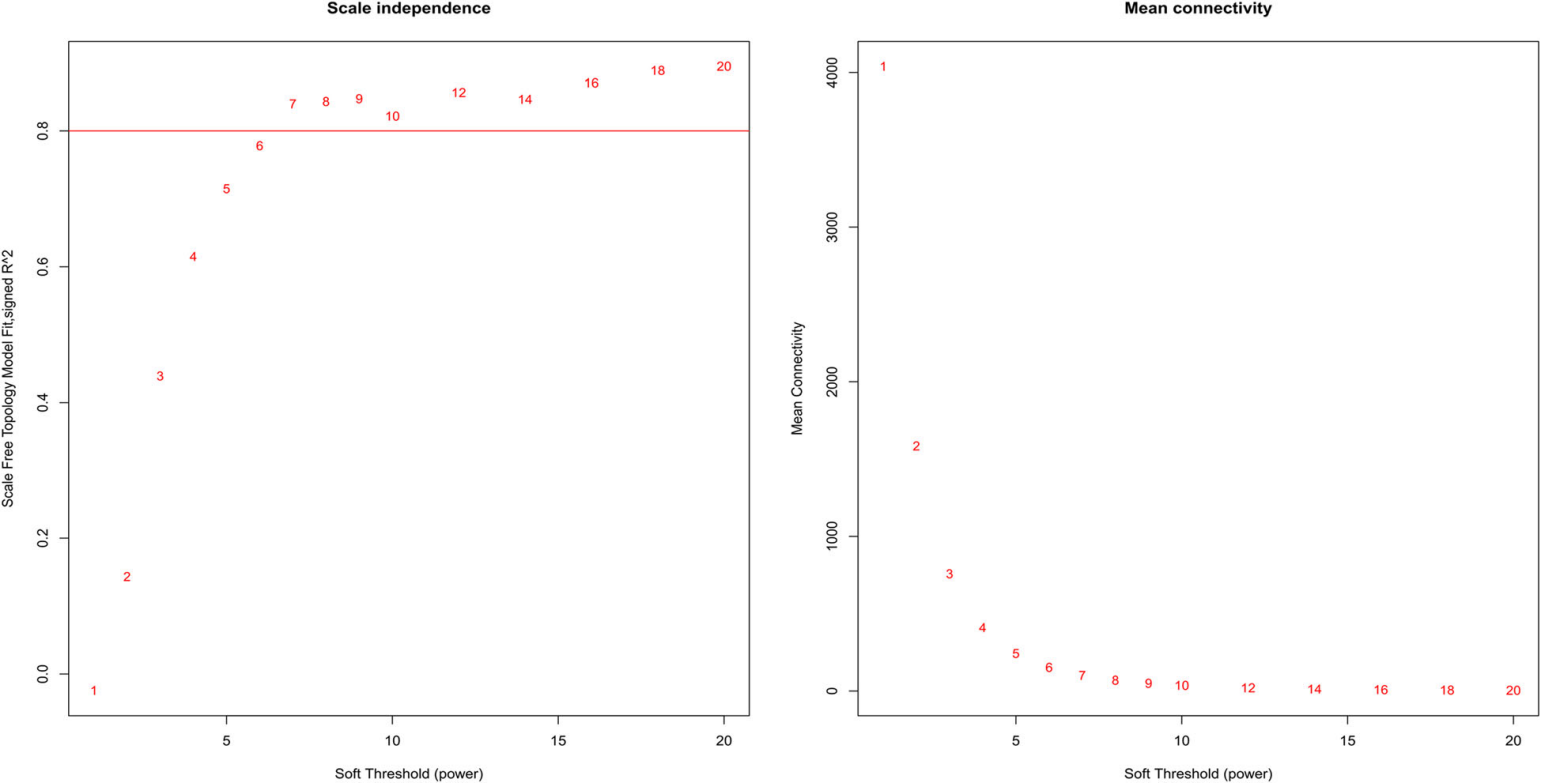
Analysis of the scale-free fit index (left panel) and mean connectivity (right panel) for various soft-thresholding powers

**Fig. 3:**
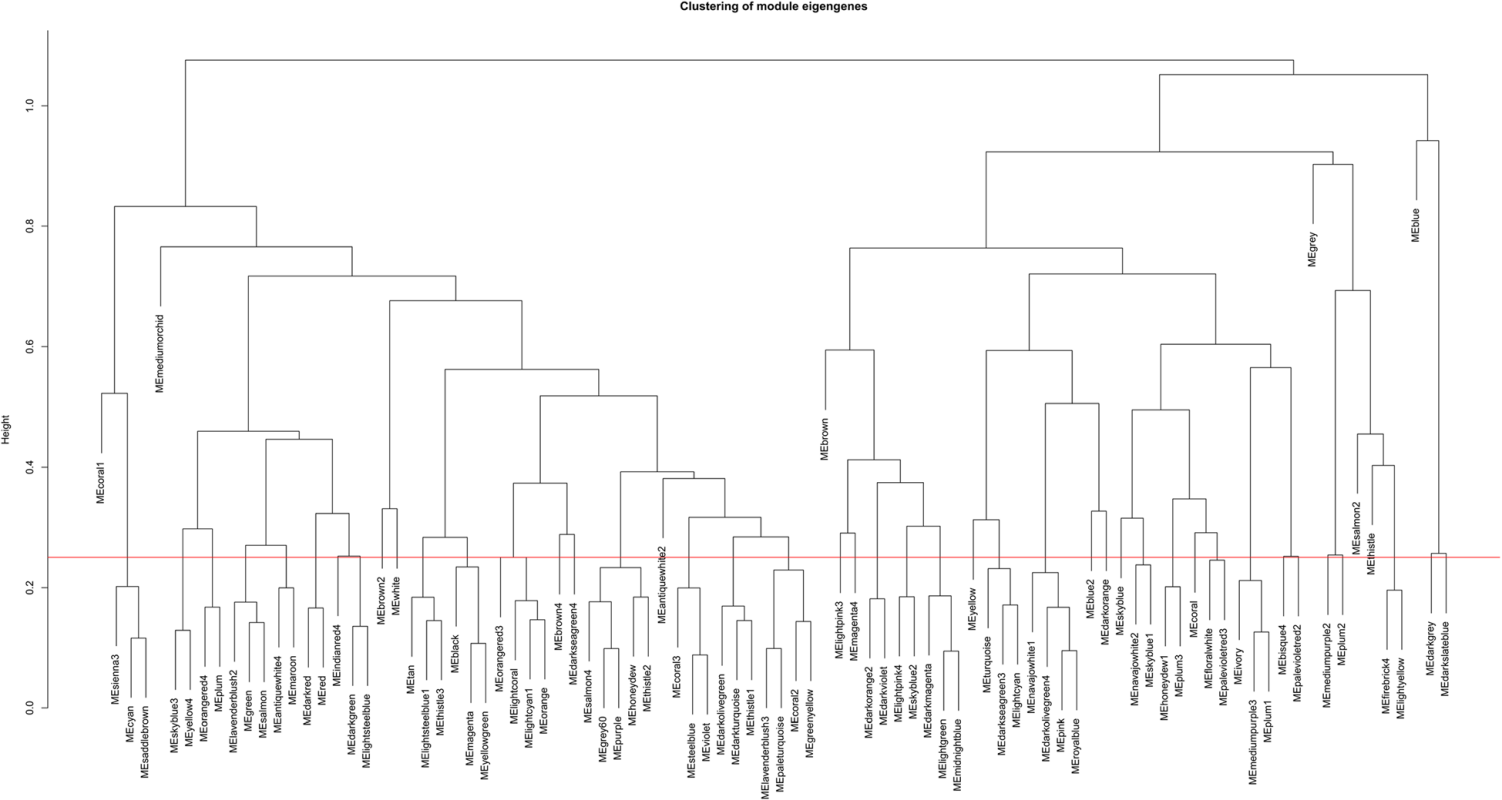
The cluster dendrogram of co-expression network modules. The red line reveals the cut-off of data filtering

**Fig. 4:**
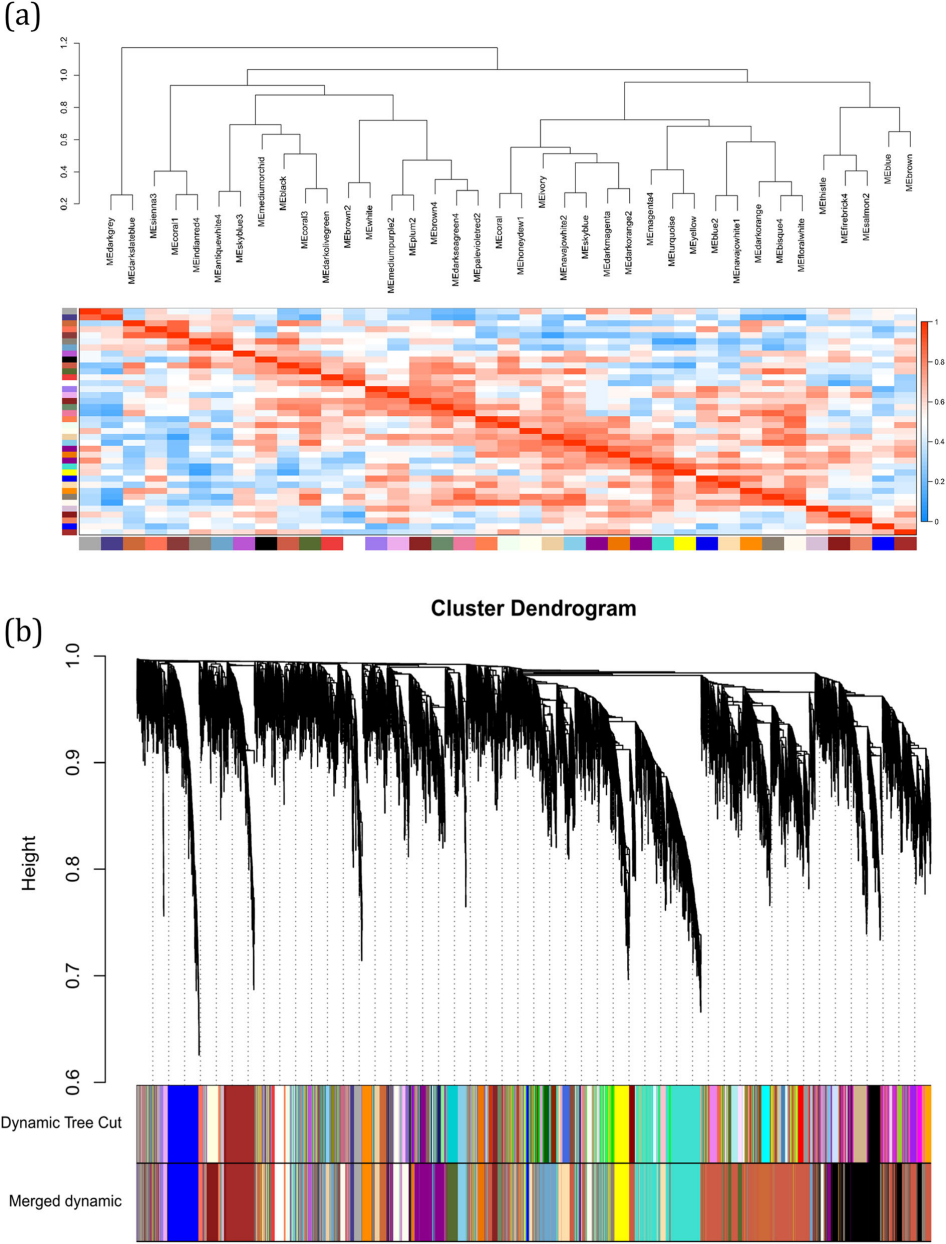
(**a**) Heatmap plot of the identified modules. Each row and column is in accordance with one module, in which red and blue shows the positive correlation and negative correlations, respectively. (**b**) Dendrogram of genes clustered based on a dissimilarity measure (1-TOM) with assigned module colors. The colored rows show the module membership obtained by the dynamic tree cut method and after merging modules

**Fig. 5:**
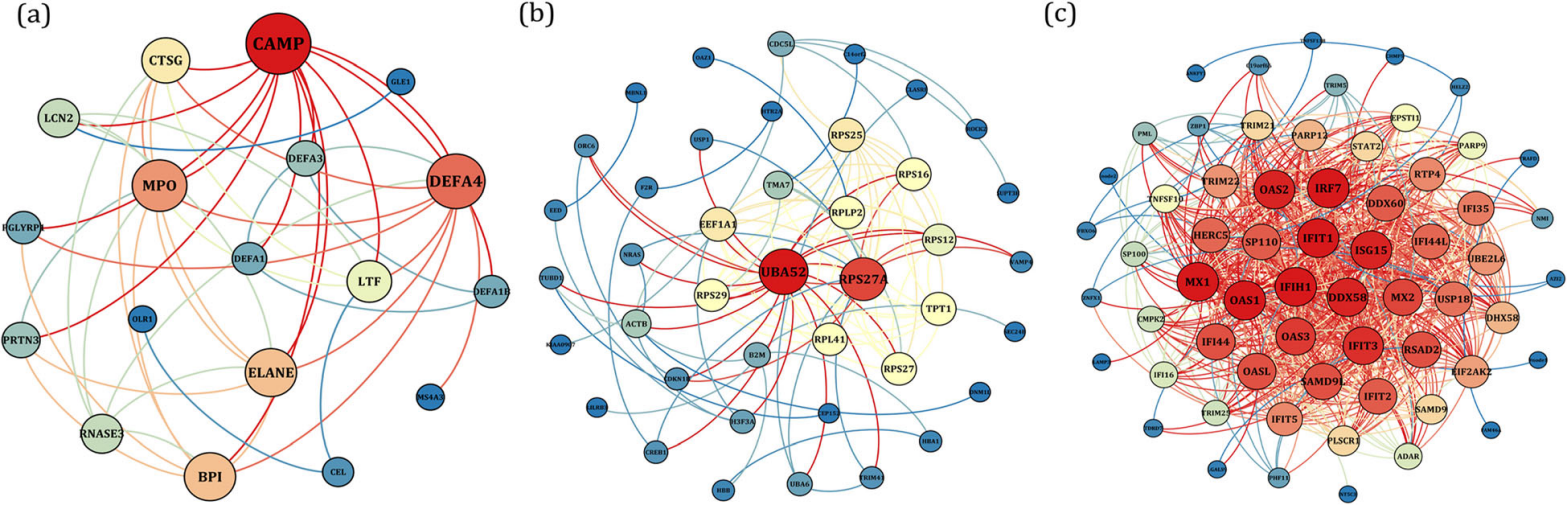
The PPINs of (**a**) mediumpurple2 (**b**) skyblue, and (**c**) yellow modules. The node size and color represent the degree level so that the higher degree is specified by the red color and bigger size

To exploration the required criterion for WGCNA, the scale-free topology fit index was calculated for various soft-thresholding power. As Fig. 2 shows, it reaches 0.84 for a power of 7 while a relatively high mean connectivity remained. This value was then applied to measure the adjacency and topological overlap matrixes. To specify the groups of co-regulated genes identified as modules, the dynamic cut-tree algorithm was utilized (Fig. 3). After merging the similar modules by applying a threshold of 0.25, 38 modules were identified to further analysis (Fig. 4a). Each row and column of the heatmap plot is in accordance with one module, in which red denotes positive correlation and blue reveals a negative correlation. Figure 4b shows the dendrogram and dynamic cut tree before and after merging modules. To determine the biologically meaningful modules, all modules were submitted into the STRING and ones which their proteins were highly connected, were selected. Figure 5 shows the protein-protein interaction networks (PPINs) of each module, in which mediumpurple2 has 18 nodes and 46 edges, skyblue has 41 nodes and 107 edges, and yellow has 60 nodes and 689 edges. The size and color of each node illustrate the degree value in which red and blue colors indicate the higher and lower degree, respectively. The chosen modules were under scrutiny in terms of gene ontology and pathway enrichment. The most related modules to the infection with influenza were identified as mediumpurple2, skyblue, and yellow. Table 1 demonstrates the genes in each module.

**Table 1:** The genes involved in each module and identified DEGs.

Modules	Genes
mediumpurple2	ELANE, DEFA3, PRTN3, DEFA1, DEFA1B, MPO, DEFA4, BPI, CAMP, RNASE3, LTF, LCN2, CEL, GLE1, CTSG, PGLYRP1, MS4A3, OLR1
skyblue	RPS25, HBB, RPS27A, RPS27, RPLP2, RPS29, UBA52, RPS16, RPL41, UBA6, EEF1A1, TPT1, USP1, TRIM41, HTR2A, NRAS, CDKN1B, B2M, ACTB, TMA7, MBNL1, CREB1, H3F3A, DNM1L, CEP152, C14orf2, CDC5L, OAZ1, HBA1, RPS12, F2R, VAMP4, ORC6, SEC24B, LILRB3, EED, CLASRP, TUBD1, SUPT3H, KIAA0907, ROCK2
yellow	ISG15, OAS1, MX1, DDX58, IFIT1, RSAD2, IFIT3, IRF7, IFIT2, OAS2, IFI35, UBE2L6, MX2, SP100, HERC5, TRIM22, TRIM25, EIF2AK2, OASL, ADAR, STAT2, TRIM21, TRIM5, PML, IFIH1, CMPK2, IFI44, FBXO6, ZBP1, IFI44L, DDX60, OAS3, IFIT5, SAMD9, SAMD9L, DHX58, RTP4, PARP12, SP110, PLSCR1, HELZ2, AZI2, IFI16, PARP9, TNFSF10, TDRD7, PHF11, TNFSF13B, EPSTI1, ANKFY1, ZNFX1, LGALS9, FAM46A, USP18, NT5C3, NMI, TRAFD1, C19orf66, CHMP5, LAMP3
DEGs (between normal and infected subjects)	OTOF, IFI44L, XAF1, IFI6, EPSTI1, IFI44, SAMD9L, EIF2AK2, IFI35, OAS3, HESX1, OASL, STAT1, ISG15, MX1, DDX60, PARP9, RNF213, IFITM3, LY6E, HELZ2, FBXO18, IRF7, ADAR, RSAD2, MOV10, IFIH1, IFIT3, STAT2, FBXO6, SAMD9, SCO2, CMPK2, NEXN, H1F0, TNK2, C2, OAS2, MYOF, SP100, DHX58, CMTR1, RTP4, PARP10, SHISA5, SLC26A8, IFIT1, SPATS2L, OAS1, RNF31, GPR84, RPA4, LOC100419583, TAP1, PML, SLC24A4, IFITM1, LGALS3BP, UBE2L6, FCER1A, TOMM7, MASTL, MS4A4A, RPL13P5, SSH1, REC8, MEF2A, BST2, PHF11, ABCD1, MX2, JAK2, IL15RA, SP110, PARP12, DEFB1, SLC1A3, CCR3, BMX, TAPBP, LENG8, PLSCR1, HERC5, HIST2H2AA3, TRIM5, IRF9, PARP14, SERPING1, BATF2, TOR1B, STAB1, RTN2, PNKD, LAP3, IL37, STRADA, N4BP1, NR2C2, EEF1B2, CD1C, RN7SK, CLEC5A, GBA, LYG1, HP, GRN, CC2D2B, FCGR1B, CARD16, NCOA7, ZBP1, EPB41L3, IFI27, TRIM22, ARHGEF11, KIAA1958, MARK3, C1QC, TRIM25, MT1G, RPL7, TMPPE, MT2A, HIST2H2AC, ADRA1D, TDRD7, ZNFX1, LTB4R, ARG1, LDLR, CAPN3, SLC30A1, BRSK1, GPD2, CUX1, TYMP, AGRN, MAFB, RPL14, ZNF839, RPL23, IFIT5, SUSD1, FXYD6, ECE1, TRAFD1, EIF3L, RIMBP3, RPL5, AXL, DNASE2, NT5C3A, CNIH1, RGL1, SUOX, LGALS9, YEATS2, MICB, MTHFD2, EIF3F, HLA-DRA, NELL1, DTX3L, TRIM14, ANKRD22, HOOK3, IL1RN, PIWIL4, TMEM51, CAPN2, PRAM1, TRIM6, TCTN1, SPPL2A, MTF1, RPL37A, FCGR1A, NTNG2, AIM2, CTSL, GORASP1, CCR1, C3AR1, ATP8B4, EEF1G, ZCCHC2, REXO1, SLC9A1, SOCS3, RPS27A, HTR3A, RAB27A, UBA7, PAQR6, MMP9, ZNF341, TAOK2, CEP152, EXOG, FLVCR2, CTGF, HIST1H2BD, TRIM38, MAP 3K7CL, CDC25C, SLC16A6, PAG1, HGD, VDR, TMEM62, DDX58 RPL4 TIAF1 ANKFY1 ADAM9 RPL23AP32 MT1A ADAP2 FBL FOXD4L1 IFIT2 ATF3 RPS9 TCN2 MAFG MDM2 OLIG3 CHMP5 LMNB1 DPH5 AGFG1 RIPK3 LGALS9C IFI16 MB21D1 MERTK SIGLEC16 CKAP4 DPRXP4 BAZ1A DISC1 SCARB2 CDKN1A TMEM110 WARS TXN APOL2 SNX20 GPR155 CRTAP TXNDC12 RPL31 TRIM21 EIF4G3 TET2 CTSD CXCR2P1 FFAR3 INPP4A RHOT1 C12orf57 LGALS8 MED15 SRGAP2 RPL3 ATL3 SLC9A8 TMOD3 GYG1 HVCN1 ASGR2 FAM209B GCH1 OGFR NELL2 SLC27A3 STBD1 NBEAL2 NDST1 TRIM69 RNF19A CD36 VNN1 CEPT1 LHX6 SLC22A18 TBC1D2 HIST2H2AB ITIH4 GLS PTP4A1 MYD88 RPL13A RPS14 PLEC UGGT2 RBM43 GBP1 SYT17 FAM46A SORT1 ADAMTSL4 RPL23AP64 USP18 ATP11C C1QB ARSA EXT1 MR1 OPLAH RPS3 BCL2L11 HK3 LOXL3 MCMBP WWP2 CD177 MCEMP1 TRPM4 SLC6A12 ABCC10 RPL21 SLITRK4 CALML4 STX16 B4GALT5 ARID3A VAMP5 DNAJC5 YIPF1 DIAPH

### Differentially expressed genes

To evaluate which genes in each module probably affected the pathogenesis routes after infection by influenza virus, 719 differentially expressed genes were found between the normal and infected subjects. In the next step, the chosen modules were explored to find which DEGs were involved in them. The results disclosed the presence of *RPS27A*, *CEP152*, *TPT1*, *UBA52* in the skyblue module, and *EIF2AK2*, *C19orf66*, *PML*, *PARP12*, *TRIM22*, *CMPK2*, *SP100*, *SP110*, *TRIM21*, *CHMP5*, *IFI44L*, *IFIT5*, *FAM46A*, *ANKFY1*, *IFIT1*, *LAMP3*, *TNFSF10*, *TNFSF13B*, *OAS2*, *PLSCR1*, *LGALS9*, *UBE2L6*, *ADAR*, *RTP4*, *IFIT2*, *IFI35*, *IFI16*, *HERC5*, *STAT2*, *OAS3*, *RSAD2*, *OAS1*, *MX1*, *IRF7*, *SAMD9L*, *DDX60*, *DDX58*, *HELZ2*, *IFIH1*, *TDRD7*, *USP18*, *SAMD9*, *EPSTI1*, *ZNFX1*, *FBXO6*, *DHX58*, *TRAFD1*, *PARP9*, *TRIM25*, *ZBP1*, *OASL*, *PHF11*, *TRIM5*, *IFI44*, *ISG15*, *MX2*, and *IFIT3* in the yellow module.

### Gene ontology enrichment

To explore the biological relationship of the genes in each module, the gene ontology (GO) enrichment analysis was performed. The genes of mediumpurple2 module were enriched in different immune systems, neutrophil and leukocyte degranulation, antimicrobial humoral response, and defense response. The most related terms to the viral infection and immune system were specified from genes of yellow module which contains defense response to virus, innate immune response, defense response to other organism, response to type I interferon, response to cytokine, regulation of viral life cycle, immune response, viral process, negative regulation of viral life cycle, regulation of viral process, negative regulation of viral process, regulation of innate immune response, cellular response to virus, regulation of defense response to virus, negative regulation of viral release from host cell, and positive regulation of immune system process. As mentioned above, the genes in the mediumpurple2 and yellow modules are activated due to viral infection and the immune system provides defense against pathogenic agent. In the skyblue module, various protein targeting and localization such as co-translational protein targeting to membrane, protein targeting to ER, protein localization to endoplasmic reticulum were highlighted. Viruses usually use the complex membrane network existing in the host cell such as endoplasmic reticulum (ER) membrane to entry, replication, and assembly. Therefore, the advent of this module was expected.

### Pathway enrichment analysis

To evaluate the pathway enrichment analysis, the Kyoto Encyclopedia of Genes and Genomes (KEGG) and Reactome Pathway Database were explored. The immune pathways, Neutrophil degranulation, Antimicrobial peptides, and Defensins were enriched by genes in the mediumpurple4. This module contains genes which involved in immune response system and have antimicrobial activity.

The genes pertaining the skyblue module were enriched in Axon guidance, signaling by NOTCH2, Infectious disease, Influenza Infection, Influenza Life Cycle, Influenza Viral RNA Transcription and Replication, Viral mRNA Translation, and rRNA processing. This module contains genes which their roles have been specified in the influenza infection pathways. Notch signaling involves several functions of cellular differentiation, cell fate, and cell survival. Viruses use several mechanisms to escape innate immune antiviral responses and cause cell survival. The downregulation of CREB1, RPS27A, and UBA52 in this pathway show the immune system domination.

Eventually, the genes grouped in yellow module were enriched in the viral and immune pathways including Influenza A, RIG-I-like receptor signaling pathway, NOD-like receptor signaling pathway, Herpes simplex infection, Cytosolic sensors of pathogen-associated DNA, Immune System, Cytokine Signaling in Immune system, Interferon Signaling, and Antiviral mechanism by IFN-stimulated genes. The RIG-I-like receptors activate innate immunity and inflammation after discernment viral RNA ligands [16]. RIG-I is required for type I IFN production in response to influenza. One of the cellular defense mechanism is performed by receptors that check uncommon RNA and DNA resulted from viral infection in the cytosol. It causes the limitation in the virus replication and activation of antiviral immunity process [17].

## Discussion

In the present study, three PPI network were identified based on the WGCNA which are significantly related to the pediatric influenza infection. Functional analysis revealed that the genes in the mediumpurple2 module implicate in the immune responses through different routes, skyblue module in the protein targeting, and the yellow module in the defense and immune responses to virus. Moreover, the analysis of differentially expressed genes revealed 719 DEGs between the normal and infected subjects. Further analysis showed that most members of the yellow and some genes in the skyblue modules were among identified DEGs, so these modules were more discussed.

Children who did not experience previous exposures to influenza viruses are more vulnerable against infection and shed larger number of virus particles for a long time [18]. Moreover, the function of immune system may be different from adult-infected subjects. The initial defense and adaptive immune responses against viral infection are performed by type I IFNs (IFN-α/β) such as RTP4, GBP1, OAS1, IFI27, and IF44L [19]. They induce the IFN-stimulated genes (ISGs) through activation of the Janus kinase–signal transducer and activator of transcription pathway [20]. The influenza A viruses hinder type I IFN signaling via induction of the suppressor of cytokine signaling-3 (SOCS-3) protein [21]. As a result, the expression level of *SP110*, *OAS1*, and *IRF*-1, which are IFN-induced genes, increases.

ISG15 has been recognized as an activator of NK cells and a driver of IFN-g secretion [22]. It has common properties with other ubiquitin-like proteins (UBLs); however, its function is influenced by the innate immunity signaling pathways. The increase in the expression of ISG15 is found after type I interferon stimulation and viral infection. Similar to type I interferons, ISG15 involves in the disease tolerance against the influenza A virus infection. Indeed, ISG15 conjugation of cellular target proteins such as Mx1 and Mx2 is needed for the antiviral activities. Moreover, ISG15 conjugation of a viral protein blocks some indispensable functions [23].

*HERC5* is another IFN-induced gene that mediates ISGylation of protein targets. It is induced by Influenza infection and elevates ISGylation as an ISG15 E3 ligase [20, 24, 25]. It also functions as a critical antiviral protein against Influenza A virus by restricting IAV-NS1.

*Samd9l* is a Sam domain containing protein which has important roles during virus infection and innate immunity [26]. The expression of *Samd9l* is increased by type I interferon and its role in the control of influenza virus infection and also pathogenesis has been proposed [27].

In particular, we also identified the *C19orf66* gene which previously reported as an IFN-induced transcript that suppresses dengue virus [28]. C19orf66 named as RyDEN can constitute a complex with the indispensable cellular mRNA binding proteins, PABPC1 and LARP1, for the efficient replication of DENV. Moreover, it was proposed that C19orf66 is a new antiviral effector which has a prominent role in the inhibition of virus replication. The increase in the expression of *C19orf66* was accompanied with up-regulation of *IFIT* genes [29, 30] which can inhibit the translation initiation, bind and sequester uncapped viral RNA, and viral protein in the cytoplasm.

Another substantial gene found in this study is *HELZ2* (Helicase With Zinc Finger 2) which is an IFN stimulated gene and immediate early gene (IEG). It was reported that the HELZ2 knockout in mice causes the enhancement of the dengue virus infectivity. Also, the mediatory of *HELZ2* in the IFN antiviral response was disclosed, so that the up-regulation of the HELZ2 transcriptional and protein in the nucleus, and activation of a transcriptional program were involved. Moreover, IEGs were introduced as the biomarkers due to their influence on the host response to viral infections [31].

*EPSTI1* is an IL-28A-induced ISGs which was identified as an anti-HCV. Also, the knockdown of EPSTI1 caused the enhancement of virus. It was stated that EPSTI1 may actuate PKR promoter and induce *IFN-β*, *IFIT1*, *OAS1*, and *RNase L*, which are PKR-dependent genes and responsible for the EPSTI1-mediated antiviral activity. Therefore, EPSTI1 was presented as a proper therapeutic target to treat HCV infection [32]. In this study, the expression level of *EPSTI1* has increased in the influenza-infected subjects versus normal cases. The up-regulation of the IFIT proteins especially IFIT1 and also OAS1 may induce the overexpression of EPSTI1, so it can be considered as the biomarker or a target for designing a drug.

PHF11 is a transcriptional co-activator of IL2 and IFNG which its knock-down causes up-regulation of the pro-inflammatory chemokine IL-8, therefore the contribution of PHF11 in epidermal recovery was proposed [33]. The overexpression of PHF11 due to the infection with Japanese encephalitis virus [34], avian influenza A virus [35], and Epstein-Barr Virus [36] were reported previously. Similarly, the expression of PHF11 was increased after influenza infection which can confirm its role in the diminution of pro-inflammatory chemokine and increase the IFNG due to the infection.

IRF7 which is overexpressed due to the influenza infection regulates the antiviral response. The viral infection causes the phosphorylation and translocation of IRF7 to the nucleus and as a result the induction of expression of type-I interferons which in turn activates IRF7 transcription through STAT2 [37, 38].

EIF2AK2 is the interferon-induced dsRNA-dependent protein kinase which has a prominent role in the innate immune response to viral infection, apoptosis, cell proliferation, and differentiation. MX1 as a member MxA protein belongs to the GTPase family and suppresses the influenza virus replication via targeting the viral nucleoprotein [39]. OAS proteins family are IFN-stimulated proteins which have prominent roles in the innate immune responses. They also catalyze the synthesis of 2 ′ -5 ′ -linked oligoadenylates, which in turn cause the activation of RNAse L and degradation of viral and cellular RNAs [40]. ZBP1 has been recently identified as an innate immune sensor of influenza virus. It regulates NLRP3 inflammasome activation and induces the apoptosis, necroptosis, and pyroptosis in the influenza-infected cells [41].

The genes involved in the mediumpurple4 were enriched in the immune pathways and neutrophil degranulation. Neutrophils are granulocytes that comprise innate phagocytic cells packed with granules containing proteins with antibacterial function [42, 43]. In this study, we identified proteins belonging to three types of neutrophil granules: primary (azurophilic: ELANE, MPO, PRTN3, BPI), secondary (specific: LTF, DEFA4, DEFA1, DEFA1B), and tertiary (gelatinase: PGLYRP1) granules. These genes were upregulated in the influenza-infected subjects. The neutrophil granule proteins have antibacterial function and have key roles in the innate immune defense against bacteria. The up-regulation of neutrophil granule proteins has been reported in viral infections and RSV-stimulated neutrophils [44–46].

In the skyblue module, *UBA52* and some genes belonging to RP family were identified in consistent with the required interaction between UBA52 and RP family for virus replication. The previous study revealed that the knockdown of UBA52 in the chicken cells causes the diminution of the progeny viral titer denoting the substantial function of UBA52 in the H5N1 influenza A virus infection [47]. However, *UBA52* and most of the *RP* genes were down-regulated in this study after the influenza infection with respect to the normal cases. It can be due to the overexpression of genes involved in the defense response to virus and innate immune response including *EIF2AK2*, *C19ORF66*, *PML*, *TRIM22*, *IFI44L*, *IFIT5*, *IFIT1*, *OAS2*, *PLSCR1*, *ADAR*, *RTP4*, *IFIT2*, *IFI16*, *HERC5*, *STAT2*, *OAS3*, *RSAD2*, *OAS1*, *MX1*, *IRF7*, *DDX60*, *DDX58*, *IFIH1*, *DHX58*, *PARP9*, *TRIM25*, *OASL*, *TRIM5*, *ISG15*, *MX2*, and IFIT3. The innate immune system responses to the entry Influenza virus by members of Toll-like receptors, and RIG-I and the NOD-like receptor family. Finally, the inter-individual variation in expression of the identified differentially expressed genes was low. It is found that inter-individual variation in gene expression profiles is related to sex, age, and the time of sample collection [48, 49]. Moreover, differences in genotype, epigenetic or environmental factors can be cause of the intrinsic differences in expression patterns.

## Conclusions

Overall, the systems virology approach based on the application of WGCNA helped us to find three modules of co-expressed genes related to pediatric influenza. Moreover, SP*110*, *HERC5*, *SAMD9L*, *RTP4*, *C19orf66*, *HELZ2*, *EPSTI1*, and *PHF11* were remarkably co-expressed with other known genes which were involved in innate immune system and defense to virus. These genes and subsequently related proteins are proposed as the candidate biomarkers and also drug targeting. However, further studies are required to evaluate and confirm them.

## Data Availability

All data generated or analysed during this study are included in this published article.

## Abbreviations

DEGs: Differentially expressed genes
GEO: Gene Expression Omnibus
GO: Gene Ontology
KEGG: Kyoto Encyclopedia of Genes and Genomes
ME: Module eigengene
PPIN: Protein-protein interaction network
STRING: Search Tool for the Retrieval of Interacting Genes
TOM: Topological Overlap Matrix
WGCNA: Weighted gene co-expression network analysis
